# Malthuism and the War

**Published:** 1914-11

**Authors:** 


### Malthuism and the War.

Just now when whole countries are being depopulated by the fell hand of war, any proposition to limit reproduction would seem to meet with disfavor. Not so with Malthusians; holding as they do that suffering and poverty both for child and parent is morally wrong, they would especially discourage child-bearing at this time, and they have made a call to the women of these nations to abstain from becoming mothers until normal conditions shall again prevail. In face of this, the warring nations, looking solely to the question of replenishing population, are encouraging marriage on leave-taking of their soldiers, thus hoping, in place of the dead father left on the battlefield, there will be in course of the allotted time another life to answer his name. This appeal does not take into account the ultimate effect on the germ plasm of that potential life by the deprivation entailed on the mother and unborn child in consequence of the war. Patriotism and personal sacrifice strongly appeal to the women of every nation, and they are usually the first to respond to their country's call. But in this instance there is a higher question of right and justice to the child to be born.

On the other hand, viewing the question from a strictly eugenistic standpoint, there is another vital principle to be considered. Many of the soldiers in the present war represent the highest specialized germ plasm that has ever been attained in the history of the world! With their lives go out forever the selective results of generations of cultural progress. To save this inherent development for replenishing the war-swept countries, there is not only justification in, but the imperative necessity for, the countries' encouraging these hasty marriages. While it is true many such marriages would fail of the purpose, and while suffering and distress would probably in many instances influence the vigor of the germ plasm thus propagated, yet in no other way can this inheritance be saved if the war continues in its wholesale devastation. Thus, there is opened another moral question for the public to decide in the light and conviction of its understanding.

